# Value of Geriatric Assessment Using the G8 to Predict Postoperative Urinary Tract Infections in Patients Undergoing Radical Cystectomy

**DOI:** 10.5152/tud.2022.22069

**Published:** 2022-07-01

**Authors:** Shugo Yajima, Yasukazu Nakanishi, Yousuke Umino, Naoya Ookubo, Kenji Tanabe, Madoka Kataoka, Hitoshi Masuda

**Affiliations:** 1 Department of Urology, National Cancer Center Hospital East, Chiba, Japan

## Abstract

**Objective:**

Urinary tract infection is one of the most common and distressing complications of radical cystectomy with urinary diversion. This study aimed to elucidate the usefulness of the geriatric-8 screening tool for predicting postoperative complications, especially urinary tract infections, in patients who underwent radical cystectomy with urinary diversion.

**Material and Methods:**

Ninety-one patients with bladder cancer who underwent radical cystectomy with urinary diversion were assessed for geriatric-8 and classified into 3 groups according to their geriatric-8 score: <11 as the low score group, 11-14 as the intermediate score group, and >14 as the high score group. We retrospectively analyzed the association between geriatric-8 score and postoperative complications classified according to the Clavien-Dindo classification.

**Results:**

The median age of the patients was 75 years (interquartile range 71-80 years) and 75 (82%) were male; 41 of the patients (45%) had high geriatric-8 score (>14), 40 of the patients (44%) had intermediate geriatric-8 score (11-14), and 10 of the patients (11%) had low geriatric-8 score (< 11). In multivariate analysis, low score of geriatric-8 was independently associated with the occurrence of grade 2 or higher urinary tract infection within 30 days [odds ratio = 5.9; 95% CI = 1.2-30.3; *P* = .03], along with female [odds ratio = 6.1; 95% CI = 1.7-21.7; *P* = .006] and open surgery [odds ratio = 6.0; 95% CI = 1.8-19.6; *P* = .003].

**Conclusion:**

The geriatric-8 score may contribute to predict postoperative urinary tract infection in patients with bladder cancer who underwent radical cystectomy with urinary diversion.

**Keywords:**

Urinary tract infection; risk factor; radical cystectomy, screening tool, G8

**Main Points**

- We demonstrated the clinical significance of the geriatric 8 (G8) screening for predicting postoperative urinary tract infection (UTI) after radical cystectomy with urinary diversion.
- Low score of G8 (<11) was significantly associated with the occurrence of grade 2 or higher UTIs within 30 and 90 days postoperatively.
- In addition to low score of G8 (<11), female and open surgery were independently associated with the occurrence of grade 2 or higher UTIs.

## Introduction

Radical cystectomy (RC) with lymph node dissection and urinary diversion (UD) is a standard treatment for muscle-invasive bladder cancer and high-risk and recurrent non-muscle-invasive bladder cancer.^1^ Due to recent improvements in surgical procedures and perioperative management, treatment-related morbidity and mortality rates have decreased over the past 2 decades, but RC remains one of the most invasive urological procedures. In particular, concern has been expressed about the increased morbidity and mortality rates of this surgery in elderly patients.^2^ In particular, urinary tract infection (UTI) after RC and UD is one of the significant postoperative concerns with an incidence of 7-30% according to several large contemporary institutional cohorts.^3^

Comprehensive Geriatric Assessment (CGA) is an evaluation of the older patient, developed by geriatric medicine, to plan health care assistance.^4^ It consists of a series of tests, which can help the clinician to better understand the global health status of the patient, but it is time-consuming and a burden for the patient.^5^ Therefore, instead of CGA, several geriatric screening tools for older adults with cancer have been investigated, such as the vulnerable Elders Survey-13 and the geriatric-8 (G8).^4^ The G8, which includes 7 items from the Mini Nutritional Assessment (MNA) and an age-related item (<80 years, 80-85 years, or >85 years), is one of the most widely studied screening tools in geriatric oncology. The G8 includes items to assess not only functional status but also nursing status, neuropsychological status, malnutrition, and polypharmacy, allowing for a multifaceted assessment of the patient’s general condition. The usefulness of G8 for predicting prognosis, functional outcomes, and complications has been widely studied in various types of cancers.^6-8^ In clinical trials, G8 was shown to be a strong prognostic determinant of functional decline in activities of daily living and instrumental activities of daily living (ADL/IADL) and overall survival,^9^ and was also associated with early mortality.^10^ The simplicity of G8, which can be performed by a physician and takes less than 5 minutes, makes it very useful in daily clinical practice.

Because of the high incidence of UTI after RC with UD,^3^ and the high surgical invasiveness, postoperative UTI can be a potentially fatal complication, especially in the elderly. A study of a large cohort of patients with diabetes mellitus (DM) and chronic kidney disease has shown that those with higher severity of frailty at the beginning of follow-up carried a rising risk of UTI during follow-up.^11^ Likewise, in the postoperative setting of RC with UD, we speculated that baseline frailty of the patient is a risk factor for UTI, and that G8 might be useful in screening for frailty.

The aim of this retrospective study was to elucidate the usefulness of the G8 in patients with bladder cancer for predicting complications after RC. Among the complications, we focused on UTI, which is a particularly frequent complication. The patients with bladder cancer who had undergone both RC and preoperative G8 assessment in our institution were the subjects, and the risks of complications were analyzed statistically.

## Material and Methods

### Patient Selection and Treatment

We reviewed the medical records of consecutive 91 patients with bladder cancer who underwent G8 assessment in the preoperative clinic and RC in our department between January 2020 and January 2022. Radical cystectomy was conducted with open or robotic assistance; robot-assisted surgery was performed using a Da Vinci Xi surgical system (Intuitive Surgical Inc., Sunnyvale, Calif, USA). All patients were operated by 3 highly skilled and experienced surgeons. Patients received antibiotic prophylaxis with second-generation cephalosporin intravenously for 3 days starting on the day of surgery. Depending on the results of preoperative urine culture, antibiotic prophylaxis other than second-generation cephalosporin was administered for 3 days. Essentially, no bowel preparation was performed. This study received approval from National Cancer Center Institutional Review Board (number 2018-159). All procedures performed in this study were conducted in accordance with the ethical principles of the declaration of Helsinki. We applied Opt-out method to obtain consent for this study.

### Geriatric-8 Assessment and Data Collection

We used the Japanese version of the MNA, which is used widely in the Japanese general geriatric population,^12^ without changing it to create the Japanese version of G8. All patients included in this study were assessed for G8 by a nurse at the preoperative outpatient clinic. The G8 score ranged from 0 to 17. We classified all patients into 3 groups according to their G8 score: a score of <11 as the low score group, a score of 11-14 as the intermediate score group, and a score of >14 as the high score group.^13,14^ Baseline characteristics of the patients such as sex, age, body mass index (BMI), Eastern Cooperative Oncology Group-physical status (ECOG-PS), the American Society of Anesthesiologists physical status (ASA-PS), Charlson comorbidity index (CCI), IADL, preoperative estimated glomerular filtration rate (eGFR), preoperative serum hemoglobin, preoperative serum albumin, neoadjuvant chemotherapy, clinical T stage, surgical approaches (open or robot-assisted), type of UD (ileal conduit (IC), cutaneous ureterostomy (CU), or orthotopic neobladder (ONB)), simultaneous nephroureterectomy, operative time, estimated blood loss, blood transfusion, lymph node yield, complications according to the Clavien-Dindo Classification (CDC), length of hospital stay, and adjuvant chemotherapy were collected.

Patients who did not achieve the max IADL score were defined as “IADL decline.”^15^ Age was categorized as <80 versus ≥80 years, CCI was categorized as <2 versus ≥2;^16^ ECOG-PS was categorized as <2 versus ≥2;^17^ ASA-PS was categorized as <3 versus ≥3.^18^ The eGFR was calculated using the Japanese Society of Nephrology’s equation.^19^

### Definitions of Complications

Postoperative complications within 30 days after surgery were recorded and graded according to the CDC. Common complications, such as paralytic ileus and UTI, were defined in detail. Regarding UTIs, the incidence within 90 days after surgery was also recorded and graded according to the CDC. Prolonged oral intake was considered to be paralytic ileus of grade 2; the need for total parenteral nutrition, insertion of an ileus tube or a nasogastric tube again were considered to be paralytic ileus of grade 3a. The presence of UTI was determined by the attending physician based on the results of urine culture and clinical findings such as fever, flank pain, and images of computed tomography. UTIs requiring administration of antibiotics were classified into grade 2; complicated UTI associated with hydronephrosis needing nephrostomy, fluoroscopy-guided ureteral stent replacement or ureteral stenting were defined as grade 3a.

### Statistical Analysis

The association between the G8 score and clinical variables was examined. In addition, we evaluated the grade of concordance between the G8 result and the appearance/absence of adverse events. We conducted a sensitivity analysis dividing the included patients into those who received CU and those who did not. Furthermore, univariate and multivariate logistic regression analyses were done to identify predictive factors of patients developing UTI (CDC 2 or more) within 30 days after surgery: reduced models were generated by backward elimination of the variable with the highest P-value from each iteration.

Pearson’s chi-square test or Fisher’s exact test was performed for categorical variables. Analysis of variance (ANOVA) was used to test for significant differences between groups with Tukey–Kramer post-hoc analysis for a continuous random variable whose probabilities are described by the normal distribution with mean and standard deviation; Kruskal–Wallis ANOVA with Steel-Dwass post-hoc analysis was used for a non-normally distributed, continuous data described by median and interquartile range (IQR). The Shapiro–Wilk test was used to test sample normality. A Bonferroni correction was used to correct for multiple comparisons. To evaluate predictive factors for the risk of UTI, when the explanatory factors are continuous variables, the receiver operating characteristic curve analysis and the Youden index was used to determine the optimal cutoff value. Risks are presented as odds ratio (OR) and 95% CI. All *P *values of <.05 (2-sided) were considered statistically significant. All statistical analyses were performed with JMP 13 (SAS Institute Inc., Cary, NC, USA).

## Results

### Patients and Clinical Characteristics

The 91 patients’ clinical characteristics and perioperative outcomes, stratified by the G8 score are shown in Table 1. The median age was 75 years (IQR: 71-80 years) and 75 (82%) were male. The median value of the G8 score was 14 (IQR: 12-15). Based on the G8 score, the patients were divided into 3 groups: 41 (45%) patients had a high (>14) G8 score; 40 (44%) patients had an intermediate (11-14) G8 score; 10 (11%) patients had a low (<11) G8 score. For the following factors, there were significant differences in distribution among the 3 groups stratified by G8 score: age (*P* < .001), ECOG-PS (*P* < .001), ASA-PS (*P* = .002), BMI (*P* < .001), preoperative albumin (*P* = .002), proportion of patients who underwent CU (*P* = .03), and lymph node yield (*P* = .04), as shown in Table 1.

### Relationship Between the G8 Score and Complications

The association between postoperative complications and the G8 score is shown in Table 2. The most common postoperative complications were UTI (32%), anemia (20%), paralytic ileus (9%), enterocolitis (5%), severe hydronephrosis (4%), anastomotic leakage (3%), and small bowel obstruction (3%). Four patients (5%) were re-operated for small bowel obstruction (3/4) and iatrogenic rectal perforation after the primary operation (1/4). One patient experienced a catheter-based intervention for acute ischemic stroke, causing hemiparesis. One patient was placed in intensive care, including longitudinal fascial incisions due to postoperative acute limb compartment syndrome in the lower leg. Unscheduled readmission within 90 days of discharge occurred for 18 patients (20%). No patient died during or after the procedure. The overall 30-day and 90-day mortality were 0 and 1% (1 patient), respectively: 1 patient died on the 67th postoperative day due to the progression of bladder cancer.

**Figure 1.**
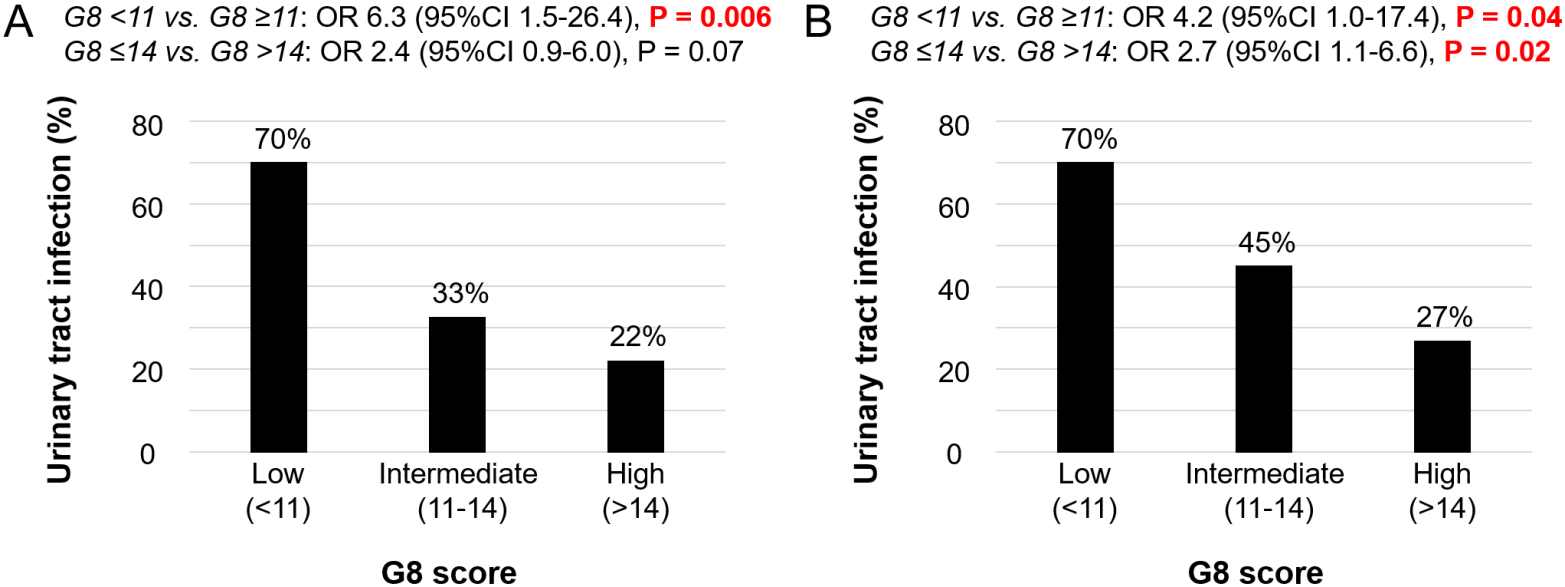
Incidence of Clavien-Dingo grade 2 or higher urinary tract infections after surgery within 30 days (A) and 90 days (B).

**Figure 2.**
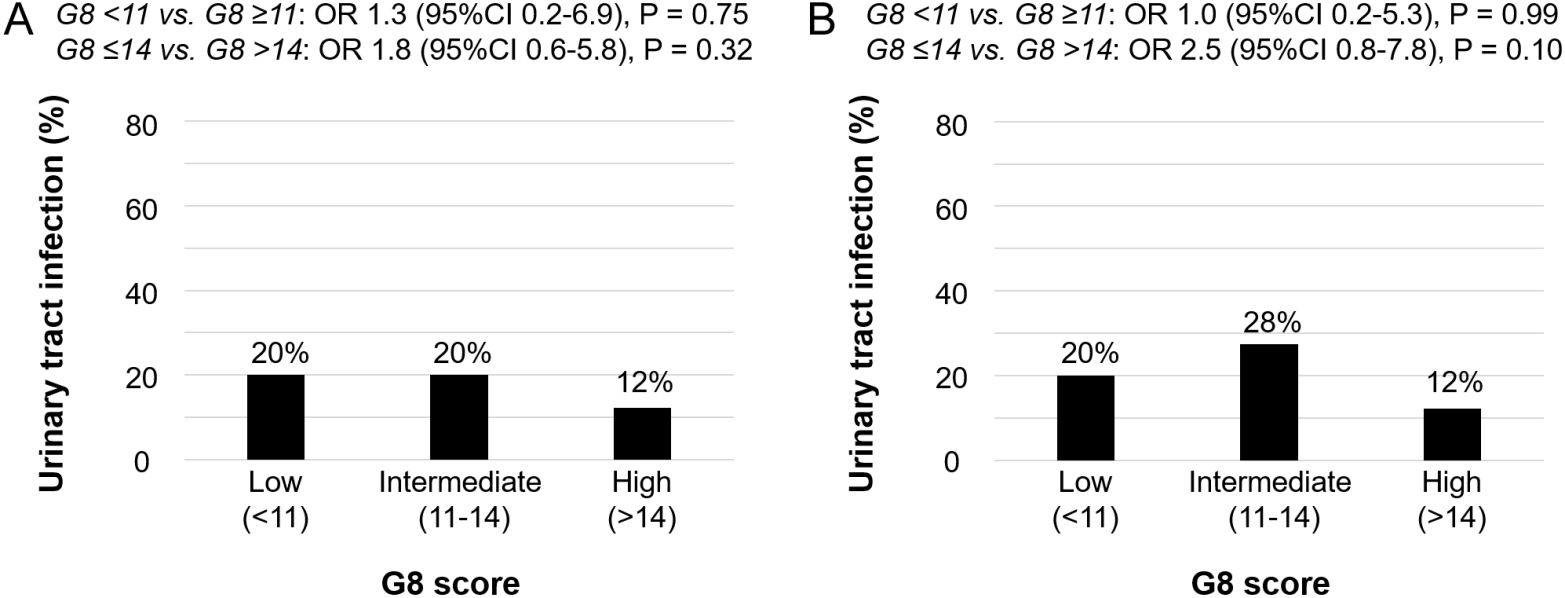
Incidence of Clavien-Dingo grade 3 or higher urinary tract infections after surgery within 30 days (A) and 90 days (B).

For the CDC grade 2 UTIs within 30 days, there were significant differences in distribution among the 3 groups stratified by G8, while CDC grade 3 UTIs were not significantly different among the 3 groups (Table 2). Figure 1 shows the distribution of UTIs for CDC grade 2 or higher (a: within 30 days, b: within 90 days), by G8 score, and Figure 2 shows the distribution of UTIs for CDC grade 3 or higher (a: within 30 days, b: within 90 days), by G8 score. Patients with low G8 scores have significantly higher rates of causing UTIs of CDC ≥2 within 30 days than patients with G8 of ≥11 (OR 6.3; 95% CI 1.5-26.4; *P* = .006), as shown in Figure 1A. Besides, patients with low G8 scores have significantly higher rates of causing UTIs of CDC ≥2 within 90 days than patients with G8 of ≥11 (OR 4.2; 95% CI 1.0-17.4; *P* = .04), as shown in Figure 1B. There was no significant association between UTIs of CDC ≥3 and G8 score, either within 30 days or within 90 days (Figure 2A and B).

### Predictors of Postoperative UTI

Among the clinical variables, for the continuous variables BMI, eGFR, preoperative hemoglobin, and preoperative albumin, the optimal cutoff values for the usefulness of the predictors of UTIs of CDC ≥2 within 30 days were 21.8 kg/m^2^, 33.0 mL/min/1.73m^2^, 12.0 g/dL, and 3.8 g/dL, respectively.

Of the clinical factors in the included patients, females (*P* = .04), low G8 score (*P* = .01), BMI of <21.8 (*P* = .02), preoperative albumin <3.8 g/dL (*P* = .048), and open surgery (*P* = .005) were significantly associated with causing UTIs of CDC ≥2 within 30 days in the univariate analysis (Table 3). In the multivariate analysis, females (OR = 6.1; 95% CI = 1.7-21.7; *P* = .006), low G8 score (OR = 5.9; 95% CI = 1.2-30.3; *P* = .03), and open surgery (OR = 6.0; 95% CI = 1.8-19.6; *P* = .003) were independent risk factors for causing UTIs of CDC ≥2 within 30 days (Table 3).

**Figure 3.**
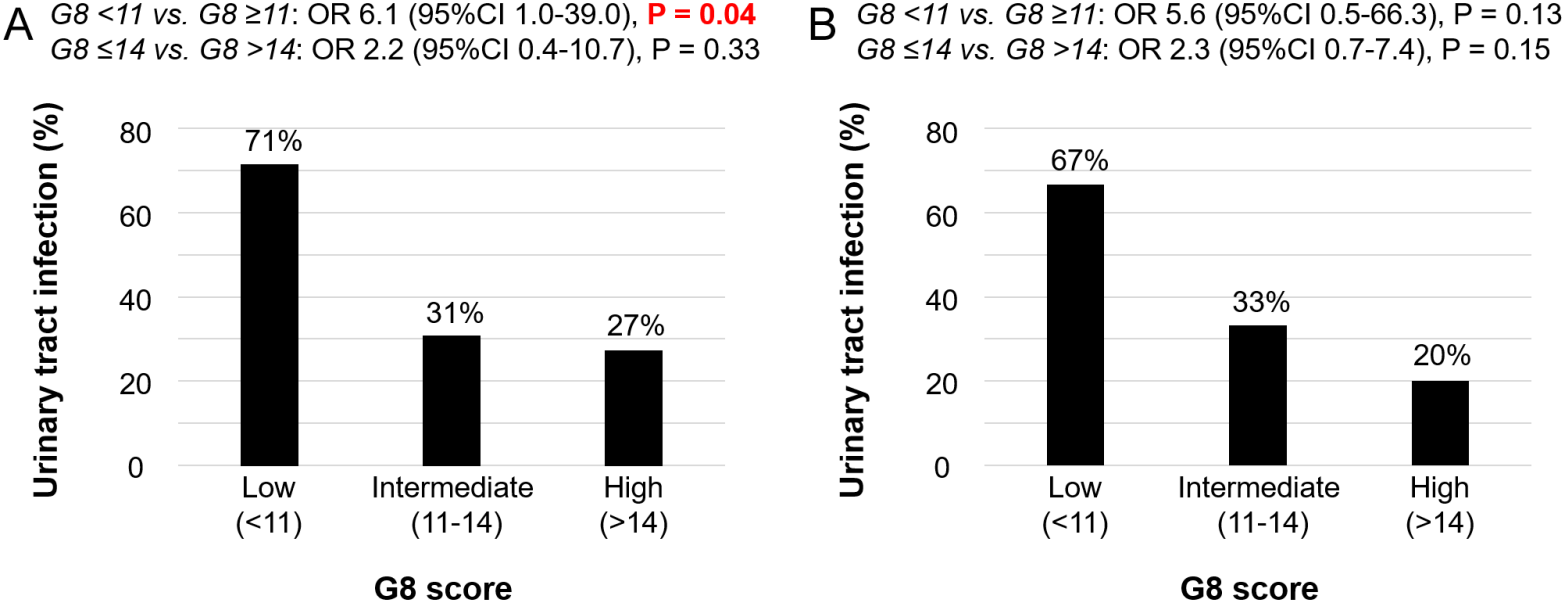
Incidence of Clavien-Dingo grade 2 or higher urinary tract infections after surgery within 30 days in the group that received cutaneous ureterostomy (A) and that not received cutaneous ureterostomy (B).

In a sensitivity analysis of the CU-received and non-CU-received groups, low G8 score (OR = 6.1; 95% CI = 1.0-39.0; *P* = .04) was significantly associated with the occurrence of UTIs of CDC ≥2 within 30 days in the CU-received group (Figure 3A). In contrast, in non-CU-received group, low G8 score (OR = 5.6; 95% CI = 0.5-66.3; *P* = .13) was not significantly associated with the occurrence of UTIs of CDC ≥2 within 30 days (Figure 3B).

## Discussion

In the present study, females, open surgery, and low G8 score (<11) were independently associated with postoperative UTI (CDC ≥2) within 30 days in patients with bladder cancer who underwent RC. 

The G8 is a frailty screening tool designed for patients with cancer, consisting of an age-related item and 7 items from the original 18-item MNA questionnaire: food intake decline, weight loss, mobility, neuropsychological problems, BMI, polypharmacy, and self-reported health.^5,6^ With total scores ranging from 0 to 17, a G8 score of 14 was conventionally suggested as the optimal cut-off value to identify vulnerable patients who should subsequently undergo a full CGA.^5,20^ The usefulness of the G8 has been shown in various cancer types to predict prognosis, complications, and functional outcomes, not only in screening before CGA.^6^ Recently, there have been reports that the novel G8 classification into 3 groups (<11, 11-14, and ≥14) has led to a more efficient identification of patients with poor prognosis than the conventional classification into 2 groups.^13,14^ In a study of elderly patients with head and neck cancer, an abnormal G8 (defined as <11) was statistically significantly associated with poor overall survival, high 30-day mortality, and high overall complication rates in a propensity score-weighted cohort.^21^ In our study, G8 was also analyzed in 3 groups, and UTIs of CDC ≥2 within 30 days were significantly higher in the patients with low G8 scores. Although there has been a report that the G8 with conventional classification could be used to predict morbidity and the severity of postoperative complications in patients who underwent RC,^22^ this is the first report that divides G8 into 3 groups and further focuses on UTI, one of the most common complications after RC.

The G8 includes items to assess not only functional status but also nursing status, neuropsychological status, and polypharmacy, allowing for a multifaceted assessment of the patient’s general condition, which may have been useful in extracting frail patients (those with low G8 scores) and predicting postoperative UTIs. In addition, G8 contains several items related to malnutrition, such as food intake decline, weight loss, and BMI, which may be useful for extracting patients with poor nutritional status. In fact, in the present study, the median preoperative albumin of the group with low G8 (<11) was 3.6 g/dL (IQR: 3.0-4.0 g/dL), which was significantly lower than their counterparts (median 4.0 g/dL, IQR: 3.9-4.2 g/dL, *P* = .007). It has been reported that patients who are classified as malnourished based on MNA scores are at higher risk of UTI,^23^ and our report is consistent with this finding. RC is one of the most invasive surgeries in urology, with a relatively high mortality and morbidity rate. Preoperative frailty diagnostic tool may allow us to identify patients at high risk for postoperative morbidity and target interventions to reduce complications to those who are most likely to benefit. Whether this new G8 subclassification can be useful for more efficient and effective medical decision making should be validated in future prospective clinical trials.

In the present report, females and open surgery were also significant predictors for postoperative UTI (CDC ≥2) within 30 days. In a study of 66 patients who underwent RC and ONB, multivariate analysis reported that female gender was a significant risk factor for predicting postoperative UTI,^24^ and the results in the current study were similar. We could not find any similar previous studies that showed that open surgery was a risk factor for postoperative UTI. Since patients who underwent open surgery tended to be older (median age 80 vs. 74, *P* = .02) and have higher CCI (median 2 vs. 1, *P* = .03) than those who underwent robot-assisted surgery, these differences in patient demographics might have resulted in more UTIs with open surgery. As a side note, previous research examining the 90-day risk of UTI in 220 bladder cancer patients who underwent RC and UD reported that type 2 DM was not a risk factor for UTI.^25^ Similarly, DM was not a risk factor for UTI in this study (OR = 0.3, *P* = .08), and there was no association between G8 score and the prevalence of DM.

Our study has several limitations. First, this retrospective study had a small sample size which was not enough for a solid analysis, thus, factors other than the G8 score may have been underestimated. The present results may not be generalizable to other institutions or patient populations, and further prospective studies using larger patient cohorts are warranted to validate our results. Then, in the sensitivity analysis of the group that did not receive CU, there was a trend toward UTI with lower G8, but it was not statistically significant. In the group that did not receive CU, there were only 3 patients with low G8 (<11), which is not a large enough sample to provide solid data. Then, we did not perform comparisons between the G8 with other validated geriatric tools, and we cannot reject the possibility that other tools screening age-related impairments might have greater accuracy to predict postoperative morbidity. Then, while our study confirms the utility of efficient screening tools to quickly detect a geriatric risk profile in the context of cancer, CGA would be needed for precise diagnosis of frailty and other geriatric syndromes. Then, this study focused only on postoperative complications and did not consider the prognosis of the patients. And finally, the determination of complications and grading had the potential for interobserver bias.

In conclusion, we have shown that the G8 classification scheme as divided into 3 subgroups is useful predictive factor for postoperative UTIs. A large-scale prospective study is warranted to further detail the value of G8 as a frailty screening tool for efficient and effective medical decision making.

## Figures and Tables

**Table 1.** Baseline and Perioperative Characteristics of the Patient Population Stratified by G8 Score

**Variables**	**Total** (n = 91)	**G8 score**			*P*	**Comparison group**	*P* * **(Post Hoc)**
Low (<11) (n = 10)	**Inter (11-14)** (n = 40)	High (>14) (n = 41)
Male, n (%)	75 (82)	8 (80)	30 (75)	37 (90)	.19		
Age, year; median (IQR)	75 (71 to 80)	86 (80 to 89)	76 (72 to 81)	73 (68 to 77)	<.001	Low versus Inter	.02
						Low versus High	<.001
Inter versus High	.04
ECOG-PS, median (IQR)	0 (0 to 1)	1 (0 to 2)	1 (0 to 1)	0 (0 to 1)	<.001	Low versus Inter	.53
						Low versus High	.003
Inter versus High	<.001
ASA-PS, median (IQR)	2 (2 to 2)	2 (2 to 3)	2 (2 to 2)	2 (2 to 2)	.002	Low versus Inter	.39
						Low versus High	.02
Inter vs High	.012
CCI, median (IQR)	1 (0 to 2)	2 (1 to 2)	1 (0 to 2)	1 (0 to 2)	.39		
Decline in IADL from baseline, median (IQR)	0 (−1 to 0)	−1 (−3 to 0)	0 (−2 to 0)	0 (−1 to 0)	.06		
BMI, kg/m^2^, mean ± SD	22.6 ± 3.1	20.8 ± 2.9	21.3 ± 2.4	24.3 ± 2.9	<.001	Low versus Inter	.86
						Low versus High	.001
Inter versus High	<.001
Preoperative eGFR, mean ± SD	53.4 ± 14.7	52.3 ± 17.3	51.7 ± 14.9	55.3 ± 14.1	.53		
Preoperative hemoglobin, g/dL, median (IQR)	11.8 (10.6 to 13.2)	11.1 (10.1 to 12.0)	11.7 (10.3 to 12.7)	12.3 (10.8 to 14.1)	.11		
Preoperative albumin, g/dL, median (IQR)	4.0 (3.8 to 4.2)	3.7 (3.0 to 4.0)	3.9 (3.8 to 4.2)	4.1 (3.9 to 4.4)	.002	Low versus Inter	.11
						Low versus High	.006
Inter versus High	.04
Neoadjuvant chemotherapy, n (%)	34 (37)	1 (10)	13 (33)	20 (49)	.053		
Clinical T stage, n (%)							
<T1	31 (37)	1 (10)	18 (46)	12 (31)	.08		
T2	32 (35)	5 (50)	12 (31)	15 (38)	.48		
T3	14 (15)	2 (20)	6 (15)	6 (15)	.91		
T4	11 (12)	2 (20)	3 (8)	6 (15)	.44		
Robot-assisted surgery, n (%)	71 (78)	5 (50)	32 (80)	34 (83)	.07		
Type of urinary diversion							
Cutaneous ureterostomy	31 (34)	7 (70)	13 (33)	11 (27)	.03	Low versus Inter	.03
						Low versus High	.010
Inter versus High	.58
Ileal conduit	52 (57)	3 (30)	25 (63)	24 (59)	.17		
Orthotopic neobladder	8 (9)	0	2 (5)	6 (15)	.18		
Simultaneous nephroureterectomy; n (%)	8 (9)	1 (10)	3 (8)	4 (10)	.93		
Operative time, minutes; mean ± SD	381 ± 78	341 ± 104	384 ± 72	389 ± 74	.22		
Estimated blood loss, mL, median (IQR)	377 (148 to 657)	451 (134 to 1010)	368 (163 to 646)	407 (141 to 692)	.96		
Transfusions, n (%)	20 (22)	5 (50)	8 (20)	7 (17)	.07		
Lymph node yield, median (IQR)	14 (9 to 19)	9 (0 to 12)	14 (8 to 20)	15 (10 to 19)	.04	Low versus Inter	.11
						Low versus High	.03
Inter versus High	.73
Length of hospital stay, days, median (IQR)	28 (23 to 35)	30 (24 to 55)	29 (25 to 36)	28 (21 to 33)	.09		
Adjuvant chemotherapy, n (%)	10 (11)	0	4 (10)	6 (15)	.40		

^*^ *P *< .017 is statistically significant in accordance with Bonferroni correction.

G8, geriatric 8; ECOG-PS, Eastern Cooperative Oncology Group Performance Status; ASA-PS American Society of Anesthesiologists physical status; IQR, interquartile range; CCI, Charlson Comorbidity index; IADL, instrumental activities of daily living; BMI, body mass index; SD, standard deviation; eGFR, estimated glomerular filtration rate.

**Table 2.** Thirty-Day Postoperative Complications Stratified by G8 Score

**Complications**	**Total** (n = 91)	**G8 score**			*P*	**Comparison Group**	*P* * **(Post Hoc)**
Low (<11) (n = 10)	**Inter (11-14)** (n = 40)	High (>14) (n = 41)
**Genitourinary**							
Urinary tract infection, n (%)	29 (32)						
*CDC grade 2*	14 (15)	5 (50)	5 (13)	4 (10)	.005	Low versus Inter	.008
						Low versus High	.003
Inter versus High	.69
*CDC grade 3*	15 (16)	2 (20)	8 (20)	5 (12)	.61		
Severe hydronephrosis, n (%)	4 (4)						
*CDC grade 3*	4 (4)	1 (10)	1 (3)	2 (5)	.57		
Anastomotic leakage, n (%)	3 (3)						
*CDC grade 2*	3 (3)	0	1 (3)	2 (5)	.69		
**Gastrointestinal**							
Paralytic ileus, n (%)	8 (9)						
*CDC grade 2*	5 (5)	0	1 (3)	4 (10)	.26		
*CDC grade 3*	3 (3)	0	1 (3)	2 (5)	.69		
Small bowel obstruction, n (%)	3 (3)						
*CDC grade 3*	3 (3)	0	2 (5)	1 (2)	.67		
Enterocolitis, n (%)	4 (5)						
*CDC grade 2*	3 (3)	0	2 (5)	1 (2)	.67		
*CDC grade3*	1 (1)	0	1 (3)	0	.52		
Rectal perforation, n (%)	1 (1)						
*CDC grade 4*	1 (1)	1 (10)	0	0	.02	Low versus Inter	.04
						Low versus High	.04
Inter versus High	N/A
Gastroduodenal ulcer, n (%)	2 (2)						
*CDC grade 2*	1 (1)	0	0	1 (2)	.54		
*CDC grade 3*	1 (1)	0	1 (3)	0	.52		
**Wound**							
Surgical site infection, n (%)	2 (2)						
*CDC grade 2*	2 (2)	1 (10)	1 (3)	0	.15		
**Cardiovascular**							
Anemia, n (%)	18 (20)						
*CDC grade 2*	18 (20)	1 (10)	7 (18)	10 (24)	.53		
Paroxysmal tachycardia, n (%)							
*CDC grade 2*	2 (2)	1 (10)	1 (3)	0	.15		
**Thromboembolic**							
Cerebral infarction, n (%)							
*CDC grade 4*	1 (1)	0	1 (3)	0	.52		
**Musculoskeletal**							
Compartment syndrome, n (%)							
*CDC grade 4*	1 (1)	0	1 (3)	0	.52		

^*^ *P *< .017 is statistically significant in accordance with Bonferroni correction.

G8, geriatric 8, CDC, Clavien-Dindo classification.

**Table 3.** Univariate and Multivariate Analysis of Risk Factors for Urinary Tract Infection (CDC Grade 2 or more) Within 30 days After Surgery

**Variables**	Univariate Analysis		Multivariate Analysis	
**OR (95% CI)**	*P*	**OR (95% CI)**	*P*
Female versus Male (ref)	3.5 (1.2-10.8)	.04	6.1 (1.7-21.7)	.006
Age, ≥80 versus <80 (ref)	2.5 (1.0-6.2)	.06		
ECOG-PS, ≥2 versus <2 (ref)	2.2 (0.3-16.6)	.59		
CCI, ≥2 versus <2 (ref)	1.1 (0.5-2.7)	.82		
IADL, decline vs independent (ref)	1.3 (0.5-3.2)	.64		
G8 score	-	-	-	-
Low (<11) vs intermediate or high (≥11) (ref)	6.3 (1.5-26.3)	.01	5.9 (1.2-30.3)	.03
Low or intermediate (≤14) vs high (>14) (ref)	2.4 (0.9-6.0)	.08		
BMI, kg/m^2^, <21.8 versus ≥21.8 (ref)	3.0 (1.2-7.5)	.02		
Preoperative eGFR, <33 versus ≥33 (ref)	0.2 (0.03-2.0)	.26		
Preoperative hemoglobin, g/dL, <12.0 versus ≥12.0 (ref)	2.5 (1.0-6.4)	.07		
Preoperative albumin, g/dL, <3.8 versus ≥3.8 (ref)	3.0 (1.0-9.0)	.048		
Types of surgical procedures, open vs robot-assisted (ref)	4.8 (1.7-13.6)	.005	6.0 (1.8-19.6)	.003
Type of urinary diversion	-	-	-	-
cutaneous ureterostomy versus the others (ref)	1.6 (0.6-4.0)	.35		
ilea conduit vs the others (ref)	0.9 (0.4-2.2)	.82		
orthotopic neobladder versus the others (ref)	0.3 (0.03-2.4)	.43		

CDC, Clavien-Dindo classification; OR, odds ratio; ECOG-PS, Eastern Cooperative Oncology Group Performance Status; CCI, Charlson Comorbidity index; IADL, Instrumental activities of daily living; G8, geriatric 8; BMI, body mass index; eGFR, estimated glomerular filtration rate.
